# Elevation of Intact and Proteolytic Fragments of Acute Phase Proteins Constitutes the Earliest Systemic Antiviral Response in HIV-1 Infection

**DOI:** 10.1371/journal.ppat.1000893

**Published:** 2010-05-06

**Authors:** Holger B. Kramer, Kerry J. Lavender, Li Qin, Andrea R. Stacey, Michael K. P. Liu, Katalin di Gleria, Alison Simmons, Nancy Gasper-Smith, Barton F. Haynes, Andrew J. McMichael, Persephone Borrow, Benedikt M. Kessler

**Affiliations:** 1 Henry Wellcome Building for Molecular Physiology, Nuffield Department of Clinical Medicine, University of Oxford, Oxford, Oxfordshire, United Kingdom; 2 The Edward Jenner Institute for Vaccine Research, University of Oxford, Compton, Berkshire, United Kingdom; 3 Medical Research Council Human Immunology Unit, Weatherall Institute of Molecular Medicine, Nuffield Department of Clinical Medicine, University of Oxford, Oxford, Oxfordshire, United Kingdom; 4 AIDS Vaccine Center, Duke University, Durham, North Carolina, United States of America; 5 Statistical Center for HIV/AIDS Research & Prevention, Vaccine and Infectious Disease Institute, Fred Hutchinson Cancer Research Center, Seattle, Washington, United States of America; NIH/NIAID, United States of America

## Abstract

The earliest immune responses activated in acute human immunodeficiency virus type 1 infection (AHI) exert a critical influence on subsequent virus spread or containment. During this time frame, components of the innate immune system such as macrophages and DCs, NK cells, β-defensins, complement and other anti-microbial factors, which have all been implicated in modulating HIV infection, may play particularly important roles. A proteomics-based screen was performed on a cohort from whom samples were available at time points prior to the earliest positive HIV detection. The ability of selected factors found to be elevated in the plasma during AHI to inhibit HIV-1 replication was analyzed using *in vitro* PBMC and DC infection models. Analysis of unique plasma donor panels spanning the eclipse and viral expansion phases revealed very early alterations in plasma proteins in AHI. Induction of acute phase protein serum amyloid A (A-SAA) occurred as early as 5–7 days prior to the first detection of plasma viral RNA, considerably prior to any elevation in systemic cytokine levels. Furthermore, a proteolytic fragment of alpha–1-antitrypsin (AAT), termed virus inhibitory peptide (VIRIP), was observed in plasma coincident with viremia. Both A-SAA and VIRIP have anti-viral activity *in vitro* and quantitation of their plasma levels indicated that circulating concentrations are likely to be within the range of their inhibitory activity. Our results provide evidence for a first wave of host anti-viral defense occurring in the eclipse phase of AHI prior to systemic activation of other immune responses. Insights gained into the mechanism of action of acute-phase reactants and other innate molecules against HIV and how they are induced could be exploited for the future development of more efficient prophylactic vaccine strategies.

## Author Summary

Acquired immune deficiency syndrome (AIDS) remains a major health problem worldwide, affecting predominantly the adult population in the western world and in developing countries in particular. Despite a tremendous effort to develop a cure or a vaccine that confers protection against human immunodeficiency virus (HIV-1) infection, this has not been achieved in a satisfactory manner to date. Recent research efforts have suggested that the earliest immune responses activated after exposure to the virus have an influence on virus spread, containment and disease progression. In this study, a panel of donors who provided plasma samples collected over a time-frame spanning the period before and immediately after detection of HIV-1 infection permitted an insight into the activation of the earliest systemic immune responses. We describe increases in plasma levels of acute-phase reactants and proteolytically processed fragments that have anti-viral activity *in vitro*. These inductions occur prior to detection of HIV-1 virus in the blood and before the first increases in systemic cytokine levels, which may represent the earliest systemic host antiviral response activated following infection.

## Introduction

Although human immunodeficiency virus type 1 (HIV-1) induces a chronic infection ultimately culminating in the development of an acquired immunodeficiency syndrome, it is now recognized that critical damage to the host immune system is mediated during the acute phase of infection, when an exponential burst of viral replication takes place, associated with massive depletion of the central memory CD4^+^ T cell pool [1], [2]. Prophylactic strategies to combat HIV-1 infection thus need to modulate events in the earliest stages of infection leading up to and impacting on this acute viral burst – which prompts an urgent need to understand the virus-host interactions occurring during this “window of opportunity”.

Adaptive responses are known to play an important role in containment of the acute burst of viral replication in AHI [3], but events in the earliest stages of infection are also likely to be heavily influenced by components of the innate immune system [3]. These include cellular determinants of the efficiency of viral entry into and replication within host cells such as the CCR5-delta32 allele, CCR2, CCL5 (RANTES), CX(3)CR1, CXCL12, or TRIM5, all of which can influence host resistance or susceptibility to HIV infection [3]. Interaction of virions with dendritic cells (DCs) early after virus transmission can have outcomes including virion destruction (following binding to langerin on resting Langerhans cells), efficient viral transmission to CD4+ T cells (following binding to DC-SIGN on sub-epithelial DCs), or the triggering of DCs (via interaction with TLRs) to produce cytokines/chemokines that may mediate antiviral activity, but may also drive immunopathological immune activation including cellular apoptosis [4], [5]. Genetic studies have helped to cast light on the *in vivo* importance of certain components of the innate immune system in acute/early HIV infection. These include associations between expression of certain KIRs and their cognate HLA alleles and resistance to, and/or control of HIV replication, implicating NK cells in control of HIV replication [6], [7], [8]. Furthermore, β-defensins, secreted from oral and mucosal epithelial cells appear to inhibit HIV-1 infection [9]. More recently, a peptide fragment derived from alpha–1 antitrypsin (AAT), a serine protease inhibitor and acute phase protein present in blood plasma, was shown to inhibit HIV host cell infection by blocking gp41 mediated cell entry [10]. Other natural factors exist that modulate HIV infection, such as a proteolytic product of the prostate phosphatase that is present in semen, which has the ability to dramatically enhance HIV infection [11].

Much of our current picture of events in the eclipse and earliest viremic phases of acute HIV-1 infection is derived from *in vitro* studies and work carried out in non-human primate simian immunodeficiency virus (SIV) infection models, as the critical initial stages of infection are very difficult to study in humans. The availability of plasma sample series collected over a time-frame spanning the eclipse and viral expansion phase of HIV infection provide a unique opportunity to gain insight into the systemic activation of immune responses during this time. Previous reports have quantified an array of cytokines and markers of apoptosis in plasma panels and described a massive systemic “cytokine storm” occurring during the viral ramp-up phase, associated with an increase in plasma levels of apoptotic microparticles [12], [13], [14]. Importantly however, no systemic elevation in apoptosis markers or cytokine levels was detected during the eclipse phase when virus is being amplified at local infection sites prior to systemic dissemination. In this study, we used a proteomics-based approach combined with biochemical and cell biological assays to characterize factors that are elevated in plasma during the earliest stages of acute HIV-1 infection in humans. We describe increases in plasma levels of acute-phase reactants and proteolytically processed fragments that have anti-HIV activity during the eclipse phase prior to detection of HIV viremia or the first increases in systemic cytokine levels, which may represent the earliest systemic host antiviral response activated following infection.

## Results

### Elevated plasma levels of acute phase markers in AHI prior to detection of viremia

**Figure 1:**
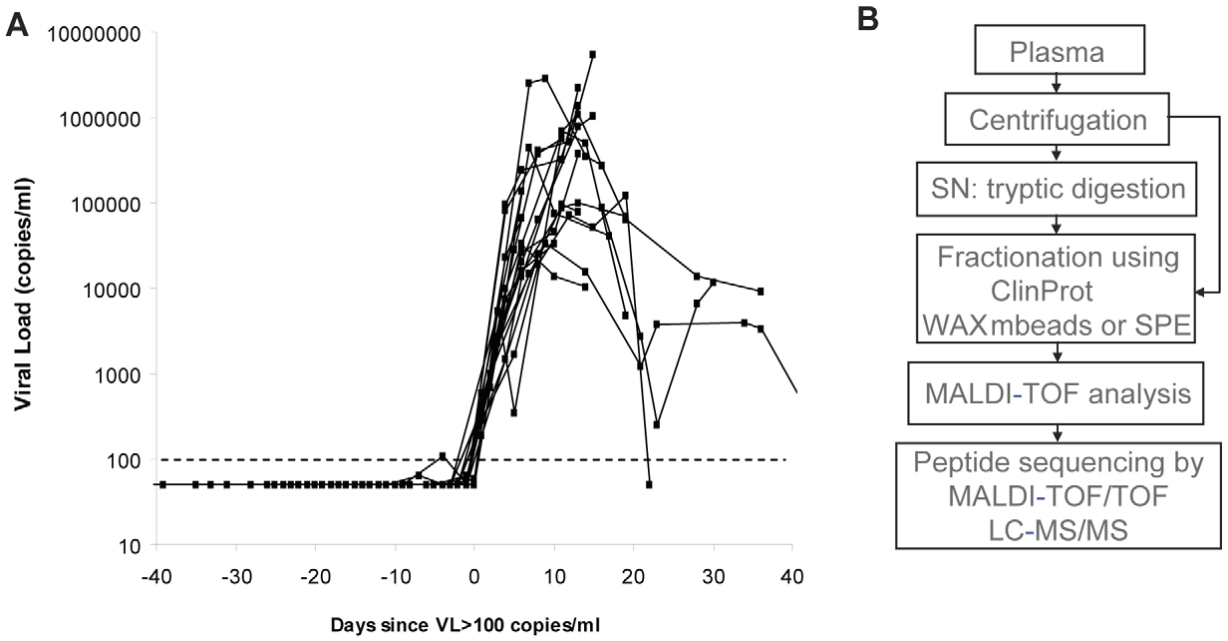
Characterization of plasma panels obtained from HIV-1-infected subjects. (**A**) Serial plasma HIV-1 RNA titers in the 19 US plasma donors acquiring HIV infection used in this study. The sample time courses are aligned relative to a common time origin, T_0_, defined as the time point when the plasma viral load first reached 100 RNA copies ml^−1^ as described in [14]. (**B**) Scheme representing the strategy for plasma sample preparation and analysis by mass spectrometry.

Samples collected at sequential time points spanning the eclipse and viral ramp-up phases from 19 US plasma donors who acquired HIV-1 infection were studied to gain insight into the kinetics of the earliest systemic anti-viral defenses activated in the acute phase of infection. Plasma was typically obtained from each donor at intervals of 2–5 d. Plasma panels were tested for HIV-1 by RT-PCR analysis of viral RNA titers, and time courses from different donors were aligned relative to the time point (T_0_) when viremia first reached levels detectable by conventional assays (>100 RNA copies ml^−1^; **Fig. 1A**). Most panels covered a time-frame from around d −20 to d +20 relative to T_0._ It is currently thought that the eclipse phase in HIV-1 infection is in the range of 7–10 d [12], [15] hence most panels likely included samples collected from time points prior to the acquisition of infection onwards.

**Figure 2:**
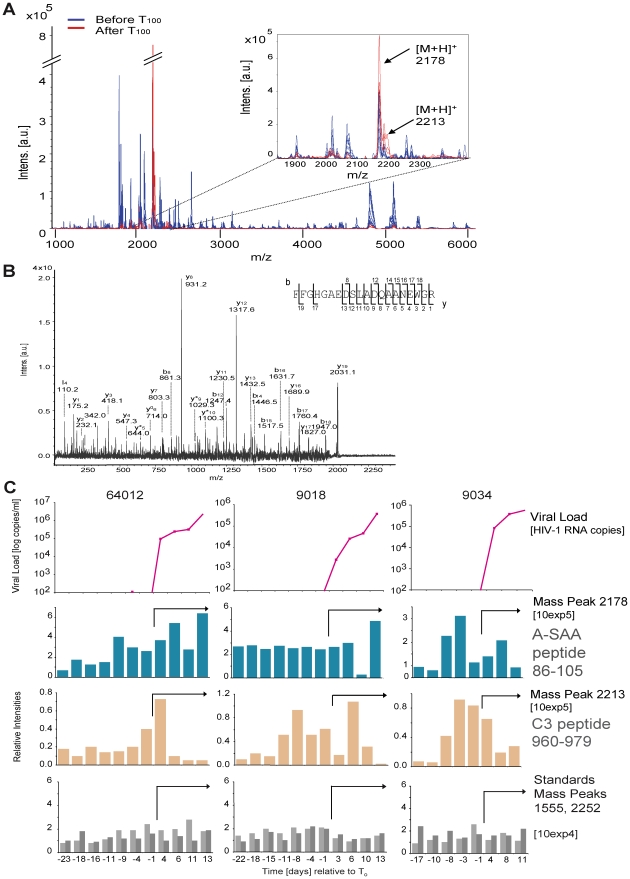
Identification of plasma proteins present at elevated levels prior to or during acute HIV-1 viremia by mass spectrometry. (**A**) Analysis of serial samples derived from plasma panel 64012 by MALDI-TOF mass spectrometry. Mass profiling reveals peaks (indicated by arrows in the insert) that are observed uniquely in HIV-1 RNA-positive (after T_0_, indicated in red, data for time points +11 and +13 days are shown) as compared to HIV-1 RNA-negative plasma samples (before T_0_, indicated in blue, data for time points −23 and −18 days are shown). (**B**) MALDI-TOF LIFT (MS/MS) spectrum of precursor ion mass 2178 Da [M+H]^+^ that was identified as the peptide 86–105 derived from human A-SAA (Swissprot accession nr. P02735). Identified b- and y- fragment ions are indicated. (**C**) MALDI-TOF-based mass profiling of three plasma panels (64012, 9018 and 9034) revealed an elevation of peaks 2178 Da (peptide 86–105 from A-SAA) and 2213 Da (peptide 960–979 from complement C3, see **Fig. S1**) before and during viremia. Viral load and T_0_ are as described previously [14]. Top panels: viral load; second and third panels: peak intensities of mass peaks 2178 Da and 2213 Da normalized to the corresponding peak intensities observed for the first time point of 64012; bottom panels: relative peak intensities of mass peaks 1555 and 2552 that were used as standards, illustrating use of comparable MS acquisition conditions for all time points. The arrows in the middle and bottom panels indicate viremic time points.

In order to determine whether there are detectable changes in plasma proteins or peptides accompanying the emergence of viremia, an initial mass spectrometry-based screen was performed on three plasma donor panels (**Fig. 1B**). Analysis of the longitudinal MALDI-TOF data revealed mass peaks that were elevated at viremic time points (**Fig. 2A**). One mass peak with a molecular mass of 2178 Dalton [M+H]^+^ was found to be considerably elevated in HIV-1-positive plasma (**Fig. 2A**). Sequencing by MALDI-TOF/TOF and LC-MS/MS identified this mass as peptide 86–105 derived from A-SAA (**Fig. 2B** and **Fig. S1A**). A semi-quantitative analysis of mass peak intensities of the 2178Da [M+H]^+^ peptide mass revealed that this peptide was elevated coincident with the increase in viremia, and in 2 of the 3 subjects, immediately prior to the detection of viremia (**Fig. 2C**), suggesting that A-SAA protein levels are elevated at these times. A second mass peak with a molecular mass of 2213 Dalton [M+H]^+^ was identified as peptide 960–979 of complement C3 (**Fig. S1B**). This peak was also elevated prior to as well as during viremia (**Fig. 2C**).

### Plasma levels of A-SAA are increased prior to and during detection of viremia in AHI

**Figure 3:**
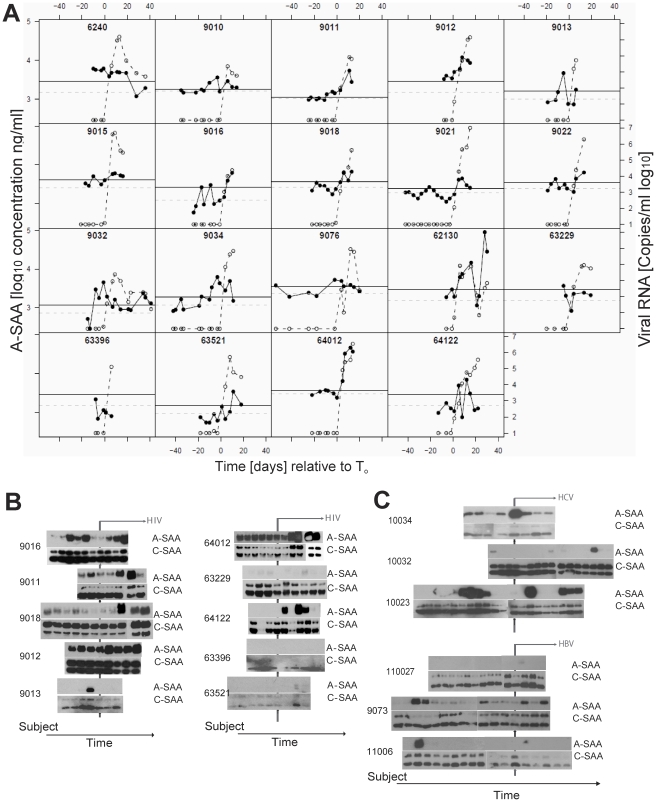
Kinetic analysis of A-SAA levels prior to and during HIV-1 viremia. (**A**) A-SAA levels as measured by ELISA in plasma panels from 19 donors acquiring HIV-1 infection. In each graph, time is plotted relative to T_0_, plasma HIV RNA levels (log_10_ copies ml^−1^) are indicated by the open circles and are as reported previously [14]. The solid circles show A-SAA protein levels. Subject-specific background levels of A-SAA (dotted grey lines) and the 90% prediction interval (threshold for defining a significant elevation, black lines) were calculated as described in the methods section. 15 out of 19 subjects show a significantly elevated A-SAA level before the first detectable viral load. (**B**). A-SAA and C-SAA protein levels in sequential plasma samples from 10 donors acquiring HIV-1 infection as assessed using anti-A-SAA and anti-C-SAA immunoblotting. The two sets of blots are positioned so that the time point when plasma viral RNA was first detected is aligned (vertical line). Post-viremic time points are indicated by the horizontal arrow. (**C**) A-SAA and C-SAA protein levels in sequential plasma samples from 3 donors acquiring HCV infection (top panel) and 3 donors acquiring HBV infection (bottom panel). As above, the two sets of blots are positioned so that the time point when plasma viral nucleic acids were first detected is aligned (vertical line); and post-viremic time points are indicated by the horizontal arrow.

A-SAA was shown previously to be elevated in patients with AIDS [16], and is commonly used as a general marker for inflammation [17], [18]. A recent study demonstrated that A-SAA has anti-viral activity *in vitro* [19]. We therefore examined a larger set of plasma donor panels (19) by ELISA to test whether elevation of A-SAA levels may be a general feature associated with acute HIV-1 infection, and how its induction is related to the increase in plasma viral RNA titers. As a control and to establish a baseline for use in statistical analysis of the data, we also measured A-SAA levels in plasma panels from five control plasma donors who did not become infected with HIV (**Fig. S2A**). Baseline levels of A-SAA (calculated as described in the methods section) varied between individuals and were generally between 600–3800ng/ml. Analysis of A-SAA levels in the plasma panels from HIV-infected donors confirmed that A-SAA was elevated relative to baseline prior to and/or concurrent within the earliest detection of viremia. Importantly, significant A-SAA elevations (i.e. falling above a 90% prediction interval) were observed prior to T_0_ (viral RNA >100 copies ml^−1^) in 15 out of 19 subjects (**Fig. 3A**). In the subject group as a whole, A-SAA levels were thus elevated significantly prior to T_0_, the time of first detection of plasma viremia (p = 0.02, as determined using a Binomial test).

To monitor alterations of the acute form (A-SAA) as well as the constitutively expressed form (C-SAA) of serum amyloid A, we performed immunoblot assays with specific antibodies (**Fig. 3B**). In 4 out of 10 plasma donor panels tested by immunoblotting (9011, 9013, 9016 and 9018), we confirmed the initial increase in A-SAA levels prior to viral ramp-up. In addition to this first wave of A-SAA induction that occurred prior to detection of viremia, immunoblotting also confirmed a second, more intense phase of elevation, observed in six of the ten donors tested (9011, 9012, 9016, 9018, 64012, 64122) that coincided with the increase in viral load. Other individuals either had very low or consistently high levels of A-SAA over the time-frame analyzed. By contrast to A-SAA, a major acute phase reactant that is inducible during the infection process, C-SAA, which was observed in an unmodified and glycosylated form, was detected with only minor inductions in all panels examined. To further explore whether elevation of A-SAA was specifically attributable to HIV-1 infection, we examined 6 plasma panels from donors who became positive either for hepatitis C virus (HCV) or hepatitis B virus (HBV) during the period of sample collection [14]. We detected increased levels of A-SAA over the time course of infection in 1/3 panels from subjects with acute HBV infection and 3/3 panels from subjects with acute HCV infection (**Fig. 3C** and **Fig. S2 B** and **C**), indicating that A-SAA induction may represent a common host response to microbial infection.

### Detection of AAT-derived VIRIP in AHI

**Figure 4:**
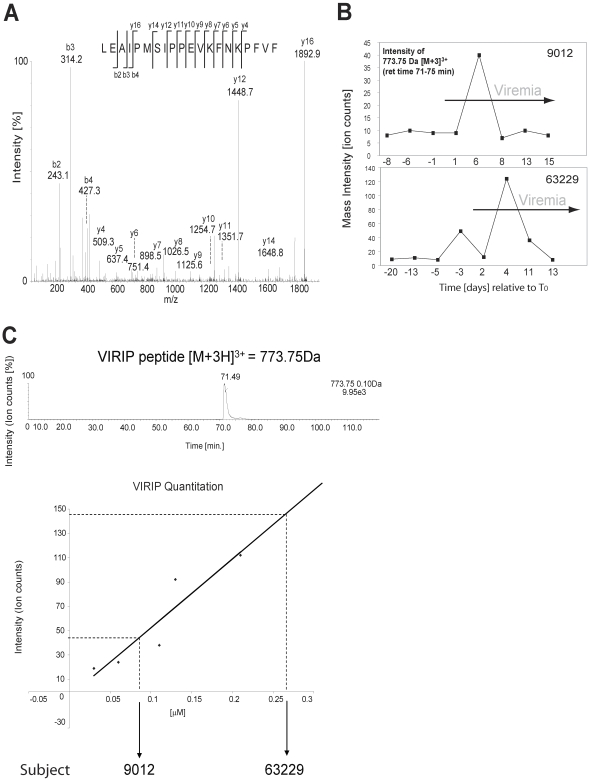
Tandem mass spectrometry-based detection of the antiviral peptide VIRIP, derived from the C-terminus of AAT, in plasma during AHI. (**A**) MS/MS spectrum of precursor ion peptide 773.75 Da [M(ox)+3H]^3+^ identified as VIRIP 377–396 derived from AAT (Swissprot accession nr P01009) as detected in plasma panel 9012 (time point 5 d post-T_0_). Identified b- and y- fragment ions are shown. (**B**) LC-MS/MS based detection of the VIRIP precursor ion 773.75 Da [M(ox)+3H]^3+^ in plasma panels 63229 and 9012. Mass peak ion counts are shown in black, and viremic time points are indicated by the grey arrow. (**C**) Quantitation of synthetic VIRIP peptide by LC-MS/MS indicates that the amount of VIRIP detected in plasma is in the low micromolar range.

A more comprehensive analysis by liquid chromatography tandem mass spectrometry (LC-MS/MS) revealed a number of other plasma components including complement factors, apo-lipoproteins and alpha-1-antitrypsin (AAT) that are present in plasma during AHI (**Table S1**). The factors found to be elevated in plasma during AHI included a C-terminal peptide derived from AAT, residues 377–396, referred to as VIRIP (**Table S1** and **Fig. 4A**), which was shown to inhibit HIV-1 entry into host cells by targeting the gp41 fusion peptide [10]. Seven plasma panels from HIV-infected individuals and five panels from uninfected controls were evaluated for the presence of VIRIP by tandem mass spectrometry in a semi-quantitative fashion. Ion counts detected for the precursor ion representing the expected molecular mass of VIRIP were correlated to viremia, and revealed an elevation of VIRIP coincident with and after the initial increase in viremia in two of the seven plasma donor panels from infected individuals, but none in the five controls (**Fig. 4B**). A semi-quantitative titration of VIRIP peptide by mass spectrometry indicated that the amount detected corresponds to an estimated value of 0.1–0.3µM of VIRIP in plasma at peak concentrations (**Fig. 4C**). Considering the sample loss during the isolation of VIRIP peptide from plasma, the effective VIRIP concentration will likely be in the range of low µg/ml, which is approximating the IC_50_ value at which VIRIP interferes with HIV-1 entry [10] (see also below).

### VIRIP is a proteolytic fragment of AAT generated by matrix metalloprotease (MMP)-mediated degradation

**Figure 5:**
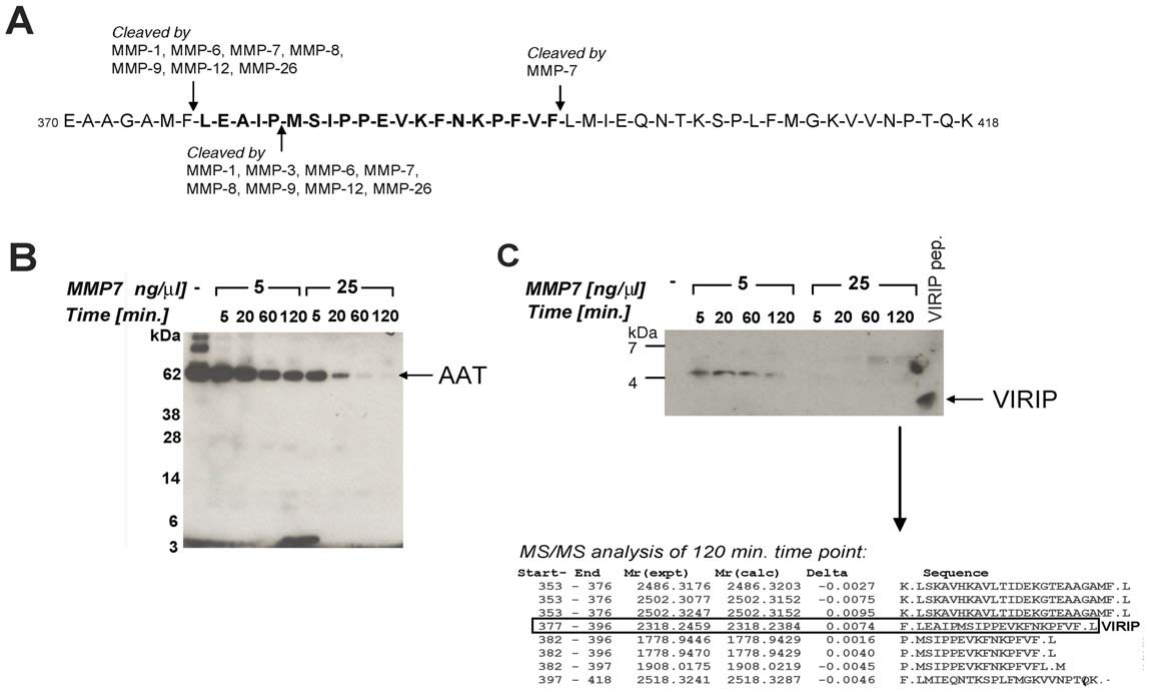
Proteolytic processing of AAT by MMP-7 generates VIRIP in vitro. (**A**) Potential MMP cleavage sites located in the C-terminal part of AAT (370–418) as predicted by the MEROPS database (http://merops.sanger.ac.uk/). (**B**) *In vitro* degradation of AAT by MMP-7 reveals distinct degradation intermediate fragments. Purified AAT was incubated with recombinant MMP-7 at the indicated concentrations and times, followed by SDS-PAGE separation on a 4–12% Bis-Tris gel and immunoblotting using an anti-VIRIP antibody. (**C**) Prolonged incubation of AAT with MMP-7 resulted in the generation of the VIRIP peptide as well as AAT C-terminal fragments; Upper panel: 16% Tris-Tricine SDS-PAGE separation followed by immunoblotting using an anti-VIRIP antibody; lower panel: LC-MS/MS analysis after 2 h digestion followed by database searching using the MASCOT algorithm identifies C-terminal AAT peptides including VIRIP (AAT 377–396) as proteolysis products.

The appearance of VIRIP in plasma samples from HIV-1-infected subjects raised the question of how proteolytic processing of AAT may be mediated under these conditions. Previous studies indicated that MMPs (collagenases and elastases) interact with and cleave AAT [20]. Inspection of the regions of AAT flanking the VIRIP sequence predicted cleavage sites for MMP-1, -6, -7, -8, -9, -12, and MMP-26 at the N-terminal, and MMP-7 at the C-terminal end of VIRIP (**Fig. 5A**). *In vitro* digestion of purified AAT with recombinant MMP-7 revealed a degradation product at 5 kDa detectable by immunoblotting consistent with the C-terminal AAT fragment 377–418 containing VIRIP (**Fig. 5B**). Subsequent analysis was carried out using LC-MS/MS, which identified peptides containing both cleavage sites required for the formation of VIRIP at Phe_376_-Leu_377_ and Phe_396_-Leu_397_ (**Fig. 5C**). A third cleavage within the VIRIP sequence at Pro_381_-Met_382_ was also observed. Importantly, formation of VIRIP itself was detected at the 2 h time point, confirming MMP-7 as a candidate protease involved in generation of the peptide *in vivo*.

### Antiviral activity of factors elevated in the plasma during AHI

**Figure 6:**
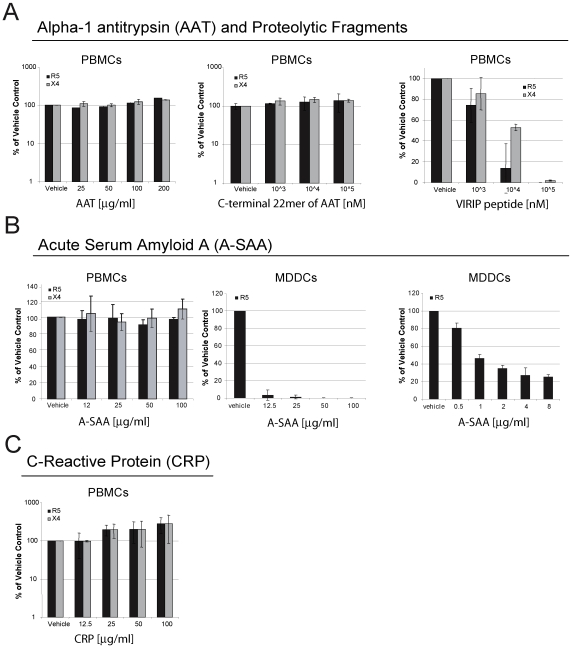
Components of the acute phase response interfere with HIV-1 infection in vitro. (**A**) Effect of AAT or its C-terminal proteolytic fragments 397–418 or 377–396 (VIRIP) on infection of PBMCs with R5- and X4-tropic viruses. HIV-1 replication was assessed by analysis of supernatant p24 levels 7 d post-infection, and results are expressed relative to p24 levels in control infected cells treated with vehicle only. R5 and X4 virus replication in PBMCs was significantly inhibited by VIRIP (p<0.0001, as determined by one-way ANOVA). (**B**) Effect of A-SAA on infection of PBMCs with R5 or X4 viruses, or infection of MDDCs with an R5 virus, assessed as in (**A**), although p24 levels in MDDC supernatants were evaluated after a 4 d rather than a 7 d infection period. Data from two MDDC infection experiments is shown, one using an A-SAA concentration range from 12.5µg–100µg/ml, and the other from 0.5µg–8µg/ml. R5 virus replication in MDDCs was significantly inhibited by A-SAA in both experiments (p<0.0001, as determined by one-way ANOVA). (**C**) Effect of CRP on infection of PBMCs with R5- or X4-tropic viruses, assessed as in (**A**). For all results, mean and STDEV values are shown for triplicates per experiment.

In order to test for potential interference with the infection process we evaluated the ability of acute phase proteins AAT, A-SAA and C-reactive protein (CRP) and the C-terminal AAT fragments 397–418 and 377–396 (VIRIP) to inhibit HIV-1 replication using *in vitro* peripheral blood mononuclear cell (PBMC) and dendritic cell (DC) infection models. PHA-activated PBMCs or monocyte-derived dendritic cells (MDDCs) were incubated with the test analytes both prior to and during infection with either an R5- or an X4-tropic virus and subsequent HIV-1 replication was monitored by the analysis of supernatant p24 levels or reverse transcriptase activity. VIRIP, derived from near the C-terminus of AAT, markedly inhibited the replication of both the R5 and the X4 virus, consistent with previous findings [10] (**Fig. 6A**). In contrast, no effect was observed with either full-length AAT or the C-terminal 22mer 398–418 (**Fig. 6A**). In addition, CRP did not inhibit the replication of either the R5 or the X4 virus in PBMCs (**Fig. 6C**). A-SAA did not exhibit any inhibitory activity in the PBMC infection system, but did inhibit the replication of the R5 virus in MDDCs as early as 12–24 h after infection (**Fig. S3**) and to a greater extent after 7 d (**Fig. 6B**). Importantly, inhibition of MDDC infection was still greater than 50% at a 1µgml^−1^ A-SAA concentration, which is well in the range of the levels detected in infected individuals (**Fig. 3A**). A-SAA was recently reported to inhibit MDDC infection by an X4/R5 dual tropic virus via down-regulation of CCR5 expression [19]. We conclude that components of the acute phase response indeed have the capacity to interfere with HIV-1 infection, thereby potentially helping to control viral dissemination in the eclipse phase.

## Discussion

The availability of sequential samples from plasma donors who became infected with HIV-1 provides a unique opportunity to study changes in plasma in the eclipse and viral expansion phases of acute infection. Recent studies described the induction of a “cytokine storm” and massive cellular apoptosis during the phase of exponential viral replication [12], [13], [14]. Here a proteomics-driven approach was used to demonstrate for the first time that acute phase proteins, some of which exhibit antiviral activity, are induced systemically even prior to the first detection of viremia and also before any detectable increase in plasma cytokine levels. The factors elevated included A-SAA, a protein primarily synthesized by cells in the liver, high-level production of which is known to be induced during the acute phase response to infection, trauma or stress by pro-inflammatory cytokines including TNF-alpha, IL-6 and IL-22 [21]. Notably, A-SAA was frequently elevated with biphasic kinetics in the plasma of subjects acquiring HIV-1 infection, an initial elevation occurring during the eclipse phase and a second elevation during the viral ramp-up phase. The latter was temporally coincident with elevations in circulating levels of multiple pro-inflammatory cytokines [14] and likely reflected a hepatic response to this systemic stimulus. The initial elevation in plasma A-SAA levels was found to occur significantly prior to detection of viral RNA in the plasma, well before any systemic elevations are detected in plasma cytokine/chemokine levels [14]. Following sexual transmission of HIV-1, virus replicates in the local genital or rectal mucosa, then spreads to the draining lymph nodes and subsequently to the gut-associated lymphoid tissue (GALT) [22]. The mechanism by which acute phase protein production is triggered during this process is currently unknown, but it may involve transfer of inductive factors to the liver (including pro-inflammatory cytokines produced at local sites of viral replication), triggering production of acute phase reactants prior to widespread virus dissemination and systemic increases in cytokine levels. Alternatively, A-SAA can be produced at extra hepatic sites: A-SAA expression has been reported in macrophages, adrenal glands, kidney and intestine, albeit at lower levels as compared to hepatocytes [17]. The initial burst of A-SAA levels may also contribute to the subsequent “cytokine storm” observed later at the systemic level, since A-SAA was shown to induce an array of immunomodulatory cytokines including of the Th1-type in monocytes, macrophages and lymphocytes [23], [24].

Activation of acute phase reactants may represent a very early line of anti-viral defense in HIV-1 infection, since A-SAA, AAT and a C-terminal peptide derived from AAT (referred to as VIRIP) were each shown to exert anti-viral activity *in vitro* [10], [19], [25], [26] (**Fig. 6A,B**). However, not all acute phase proteins that are induced in response to inflammation have anti-viral properties as was observed with CRP (**Fig. 6C**), which was also elevated systemically in plasma of some donors prior to viremia (data not shown). We found selective inhibition of HIV-1 infection of MDDCs but not PBMCs by A-SAA. Its ability to inhibit HIV-1 replication was previously proposed to be mediated by down-regulation of CCR5 expression [19]. The capacity to inhibit R5 virus replication in MDDCs but not PBMCs may thus be due to the fact that MDDCs express only low levels of CCR5, whereas CCR5 expression on CD4^+^ T cells is much higher. It is unlikely that the inhibitory effects of A-SAA are limited to specific pathogens only, as this acute phase reactant was described to be up-regulated in a number of pathological processes [17] including inflammation and diverse bacterial and viral infections [27], [28], [29]. Consistent with this, we observed marked elevation of A-SAA in plasma panels from donors acutely-infected with HCV. Notably, A-SAA has been shown to mediate antiviral activity against this viral pathogen too, an effect proposed to be mediated by mechanisms distinct from its inhibitory effect on HIV-1 infection [30], [31]. In acute HCV infection, A-SAA induction may be stimulated as a consequence of viral replication in the liver, and the associated cytokine response. In acute HBV infection little A-SAA elevation was observed. This may relate to the different replication kinetics of HBV as compared to HIV-1 and HCV, and/or the relatively muted cytokine response activated in acute HBV infection [14].

Genetic studies suggest that polymorphisms observed in AAT, another component of the acute phase response, are linked to susceptibility to HIV-1 infection [32], [33]. AAT was initially reported to block the activity of HIV-1 protease [26], [34], [35]. Subsequently, an AAT-derived 26mer C-terminal peptide was shown to inhibit HIV-1 LTR gene expression *in vitro* [36], [37]. However, the strongest effect reported so far is exerted by VIRIP generated as a proteolytic product from AAT, which inhibits viral entry by binding to the gp41 fusion peptide and is active in the low micromolar range [10]. For both A-SAA and VIRIP, we were able to detect endogenous levels in plasma that are in the range capable of mediating viral inhibition, which raises the possibility that such factors play a role in combating viral replication, particularly in the earliest stages of infection.

No strong correlation was found between the initial timing of A-SAA elevation and either R_0_ (the viral reproductive rate), the slope of viral ramp-up or the highest recorded viral load in the 19 subjects studied here. Nevertheless, the relationship between the magnitude and dynamics of early acute-phase protein production and the acute viral burst and subsequent efficiency of control of viremia should be addressed in a future study on a larger cohort from whom samples were collected over a longer time-frame extending into early infection. Interestingly, we also noted that levels of AAT proteolytic fragments in plasma from subjects chronically-infected with HIV-1 were elevated as compared to those in healthy controls (data not shown), suggesting that acute phase proteins may also play a role in chronic viral infection. It is possible that components of the acute phase response may not only contribute to the control of viral replication in infected individuals, but may also be involved in mediating resistance to infection. For instance, HIV-1-exposed uninfected individuals have been shown to have elevated levels of cleaved forms of A-SAA [19], which suggests that their anti-viral activity contributes to resistance against infection.

Evidence from the literature and our own data suggest that MMPs are responsible for AAT proteolysis, in particular MMP-7, MMP-9 and MMP-26 [38], [39]. Altered levels of MMP-9 have been reported to correlate with HIV-1 infection, and breakdown of extracellular matrix has been suggested to aid dissemination of the virus [40], [41]. Our *in vitro* experiments confirmed the known MMP-7 cleavage sites on the C-terminal part of AAT and also demonstrated that VIRIP can be generated despite presence of an additional cleavage site within the sequence of the peptide. This cleavage site between Pro_381_-Met_382_, directly neighbouring the active site Ser_383_, has been shown previously to be sensitive to the oxidation state of the Met_382_ residue [42], thereby protecting VIRIP from further degradation. In addition, our results support the notion that the biological function of cleavage of AAT by MMP-7 may not solely be inactivation of its serine protease function, but also to generate new proteolytic peptides that have additional activities in themselves.

The abundant acute phase proteins that we were able to detect elevations in during AHI may be only selected examples of constitutively-produced or inducible analytes that play a role in combating infections. There is an increasing amount of evidence suggesting that “endogenous factors” exist that have inherent inhibitory activity towards infectious pathogens [43]. These include antimicrobial polypeptides [44] and antiproteases such as cystatins that have also been detected in cervical mucosa [45]. Insights gained into the mechanism of action of innate factors and acute phase reactants against HIV-1 and how they can be induced should be considered for novel vaccine strategies and therapeutics.

## Materials and Methods

### Ethics statement

Plasmapheresis samples from the US plasma donor cohort used in this study were purchased from Zeptometrix Corporation and SeraCare Life Sciences. Donors were recruited via the SeptaCare Special Donor Program and enrolled for plasma donation after a medical examination and an interview with medical staff, in which it was explained that the donated plasma will be used by dedicated drug and vaccine researchers to perform research to help others. This study was approved by the Oxford Tropical Research Ethics committee (OXTREC, University of Oxford) and the NIH Office of Extramural Research (Nr. 201029 to B.M.K).

### Plasma samples and viral load analysis

Panels of sequential samples obtained by plasmapheresis from US plasma donors who became infected with HIV-1, HBV or HCV and control subjects were purchased from Zeptometrix Corporation and SeraCare Life Sciences and were stored at −80°C before use. Details of the panels and methods used for analysis of HIV-1, HBV and HCV viral loads are as described [14]. Each plasma panel included samples collected prior to detection of plasma viremia through to seroconversion. The plasma panels from HIV-infected individuals were temporally aligned relative to a common time origin (T_0_), defined as the time point when the viral load first reached detectable levels (>100 viral RNA copies ml^−1^), as described previously [14]. The median and range duration of observation prior to the first detectable HIV RNA level (T_0_) for the 19 panels used in this study was 21 days with a range of 7 to 58 days.

### Sample preparation for analysis by mass spectrometry

Plasma donor samples were fractionated using weak anion exchange (WAX) magnetic beads (Bruker Daltonics, Bremen, Germany) according to the manufacturer's recommendation (Text S1).

### Analysis by MALDI-TOF/TOF and LC-MS/MS tandem mass spectrometry

For analysis by MALDI-TOF/TOF, a solution of α-cyano-4-hydroxycinnamic acid (matrix, 3 mg) in ethanol∶acetone (10 ml, 2∶1) was prepared freshly. Samples processed as described above and matrix solution were mixed in a ratio of 1∶4, and 1 µL aliquots were spotted in triplicates on an Anchor Chip MALDI plate (Bruker Daltonics, Bremen, Germany). Data was acquired on an UltraFlex MALDI-TOF/TOF instrument (Bruker Daltonics, Bremen, Germany) at 60% laser power until a total intensity of 2×10^5^ ion counts was reached. Spectra were analyzed using Bruker Daltonics FlexAnalysis software (version 2.4). For LC-MS/MS tandem mass spectrometry, analysis was carried out by injection of 3 µl of sample prepared as described above on a nanoAcquity UPLC system coupled to a Waters Q-TOF Premier tandem mass spectrometer in MS^E^ (high/low collision switching) mode as described previously [46]. Processing of raw data was performed using the ProteinLynx Global Server 2.2.5 software and the data were interrogated on an in-house MASCOT server (version 2.2). Alternatively, samples were analyzed on a Bruker HCTplus Ion Trap tandem mass spectrometer (Bruker Daltonics, Bremen, Germany) as described [47]. Semi-quantitative analysis of MS data was based on measuring peak heights observed for the peptide masses that were assigned to A-SAA_86–105_ [M+H]^+^ 2178Da, C3_960–979_ [M+H]^+^ 2213Da (analysis by MALDI-TOF) and VIRIP_377–396_ [M+3H]^3+^ (methionine oxidized form) 773.75Da (analysis by LC-MS/MS). Seven plasma donor panels of infected individuals (61 time points total) and five uninfected control plasma donor panels (40 time points total) were examined. For an approximate estimation of VIRIP quantities in plasma, synthetic VIRIP was synthesized using Fmoc chemistry based solid-phase peptide synthesis on an automated synthesizer (Advanced ChemTech, Louisville, KY, USA) and different concentrations measured by LC-MS/MS by monitoring the [M+3H]^3+^ precursor ion intensities under the same conditions used for the analysis of plasma samples. It should be noted that sample loss during plasma sample processing may lead to an underestimation of the effective VIRIP concentration in plasma.

### In vitro digestion of AAT by MMP-7

Recombinant active MMP-7 (EMD Biosciences, Gibbstown, NJ) (7.2 µg, 4 µl) was added to a solution of AAT (Sigma Aldrich, St. Louis, MO) (100 µl, 2.9 µg µl^−1^) in 100 mM ammonium bicarbonate buffer and the mixture was incubated at 37°C. Aliquots (5 µl) were removed for immunoblotting at 0, 5, 20, 60 and 120 min, mixed with RSB (5 µl) and retained for analysis. Immunoblotting was carried out following gel electrophoresis on 4–12% Bis-Tris or 16% Tris–Tricine gels (Invitrogen, Carlsbad, CA) for increased resolution of low molecular weight species. LC-MS/MS analysis was carried out with the 5 min and 120 min time point samples following desalting and concentration by methanol chloroform precipitation [48].

### ELISA assays

Analysis of plasma samples using an anti-SAA ELISA assay was performed using a commercial SAA ELISA kit (Abazyme, Needham, MA) and conducted according to the manufacturer's instructions. Plasma samples were diluted with assay buffer to obtain final concentrations within the linear range of the assay of 1–80 ng ml^−1^. Inclusion of recombinant protein standards demonstrated that A-SAA was sensitively detected by the assay while C-SAA was non-reactive up to final concentrations of 750 ng ml^−1^.

### HIV-1 inhibition assays

HIV-1 infection assays used viruses derived from the infectious molecular clones pNL4.3-BaL.ecto (R5-tropic) and pNL4.3 (X4-tropic), supplied by John Kappes and Christina Jambor (University of Alabama at Birmingham, USA). Cryopreserved healthy donor PBMCs were stimulated with 2.5ug ml^−1^ PHA (Sigma Aldrich, St. Louis, MO) for 2 d in R10 medium (Invitrogen, Carlsbad, CA) and 1 d with 50U ml−^1^ IL-2 (Roche, Nutley, NJ) in R10 medium adjusted to contain 20% serum. MDDCs were generated by culturing CD14^+^ cells isolated from buffy coats (National Blood Service, London, UK) using a Human Monocyte Isolation Kit II (Miltenyi Biotec, Cologne, Germany) for 6 d in 10ng ml^−1^ IL-4 and 100ng ml^−1^ granulocyte-macrophage colony stimulating factor (GM-CSF) (eBioscience, San Diego, CA). The proportion of CD14^lo^CD11c^hi^ MDDC was typically 95%. Inhibitors included CRP (ProSpec-Tany TechnoGene LTD., Rehovot, Israel), AAT (Sigma, Gillingham, UK), A-SAA (MBL International, Woburn, MA), VIRIP and the 22 amino acid AAT fragment C-terminal to this peptide (both synthesized by Fmoc chemistry as described above). CRP, AAT and A-SAA were dissolved in serum-free OptiMEM (Invitrogen, Carlsbad, CA), VIRIP and the 22 amino acid AAT fragment were dissolved in DMSO (Sigma-Aldrich, St. Louis, MO) and diluted to a 2% solution in OptiMEM. Inhibitors were diluted into cell culture medium to give the indicated final concentrations. The final concentration of DMSO in the culture medium was never more than 0.2%. Control cultures were treated with a similar volume of 2% DMSO OptiMEM or plain OptiMEM (vehicle only) to match the addition of inhibitor. PBMCs were preincubated with inhibitors for 2 h prior to infection, then were infected with R5 or X4 virus at a MOI of 0.01 for 16 h at 37°C in the presence of inhibitor. Excess virus and inhibitor were washed out and cells were cultured for 7 d in R10 medium plus 50U ml^−1^ IL-2. Supernatants were harvested and p24 levels were determined by ELISA (Advanced BioSciences Laboratories, Inc., Kensington, MD). MDDCs were preincubated with inhibitors for 1 h prior to infection at a MOI of 0.1 for 2 h at 37°C in the presence of inhibitor; and after washout of inhibitor and excess virus, cells were cultured for 4 before reading out supernatant p24 levels as described above. Statistical analysis of the data from the viral inhibition assays was performed using a one-way ANOVA.

### Statistical analysis of ELISA data on plasma A-SAA levels

Two statistical tests were performed on transformed data from the sample time-courses from the five control non-HIV-infected plasma donors and the time points prior to D-15 in the time-courses from HIV-infected plasma donors: a Wilcoxon test for a two-group comparison (control vs. HIV-infected, p = 0.28) and an ANOVA test in a linear mixed model for the group difference (p = 0.4). As no significant differences were found between the controls and pre-D-15 data from the HIV-infected subjects, both were used to determine baseline A-SAA levels in the HIV-infected subjects. Linear mixed-effects models were fit to these data to estimate the subject-specific baseline level of A-SAA in each HIV-infected individual. A-SAA values above the 90% upper prediction bound was considered significantly elevated (see **Fig. 3A** and **S2A**). A two-sided Binomial test was conducted to examine whether the first elevation of A-SAA occurred significantly before T_0_. Additional tests of associations between the timing of first A-SAA elevation and three different parameters of viral replication (viral reproductive rate R_0_, slope of viral ramp-up and the highest recorded viral load) were also performed using linear models.

## Supporting Information

Figure S1 Identification of A-SAA and complement C3 derived peptides by tandem mass spectrometry. (A). LC-MS/MS analysis of plasma donor sample 64012 time point T_0_+4 identified precursor ion 1108.6 Da [M+2H]^2+^ that corresponded to peptide 960–979 derived from complement C3 (Swissprot accession nr. P01024). Identified b- and y- fragment ions are indicated. (B). LC-MS/MS analysis of plasma donor sample 64012 time point T_0_+13 revealed precursor ion mass of 1090.4 Da [M+2H]^2+^ that corresponded to peptide 86–105 derived from A-SAA (Swissprot accession nr. P02735).
(0.21 MB PDF)

Figure S2 A-SAA levels as measured by ELISA in plasma panels from 5 control plasma donors, 3 plasma donors acquiring HBV infection and 3 donors acquiring HCV infection. (A). A-SAA levels in plasma panels from five control non-HIV-infected plasma donors. The solid circles show A-SAA protein levels. Subject-specific background levels of A-SAA (dotted grey lines) and the 90% prediction interval (threshold for defining a significant elevation, black lines) were calculated as described in the methods section. (B). A-SAA levels in plasma panels from three HBV-infected donors. In each graph, time is plotted relative to T200 (first time point where DNA levels are above 200 copies ml^−1^). A-SAA protein levels are indicated by black squares and solid black lines and HBV DNA levels by black triangles and dotted black lines. (C). A-SAA levels in plasma panels from three HCV-infected donors. In each graph, time is plotted relative to T600 (first time point where RNA levels are above 600 copies ml^−1^). A-SAA protein levels are indicated by black squares and solid black lines and HCV RNA levels by black triangles and dotted black lines.
(0.33 MB PDF)

Figure S3 Effect of A-SAA on HIV infection of MDDCs. MDDCs were incubated with the indicated concentrations of A-SAA and infected with R5 virus produced from the infectious molecular HIV clone pNL4.3-Bal.ecto. Reverse transcriptase levels were determined at 12, 24 and 48 h. Results are the average of values from four sets of MDDCs generated from four separate buffy coats. The error bars indicate 1 standard error above and below the mean.
(0.13 MB PDF)

Table S1 Elevated proteins present in AHI plasma prior or during viremia identified by mass spectrometry
(0.03 MB XLS)

Text S1 Supporting Information
(0.03 MB DOC)

## References

[1] Li Q, Duan L, Estes JD, Ma ZM, Rourke T (2005). Peak SIV replication in resting memory CD4^+^ T cells depletes gut lamina propria CD4^+^ T cells.. Nature.

[2] Mattapallil JJ, Douek DC, Hill B, Nishimura Y, Martin M (2005). Massive infection and loss of memory CD4^+^ T cells in multiple tissues during acute SIV infection.. Nature.

[3] Deeks SG, Walker BD (2007). Human immunodeficiency virus controllers: mechanisms of durable virus control in the absence of antiretroviral therapy.. Immunity.

[4] Haynes BF, Shattock RJ (2008). Critical issues in mucosal immunity for HIV-1 vaccine development.. Journal of Allergy and Clinical Immunology.

[5] McMichael AJ, Borrow P, Tomaras GD, Goonetilleke N, Haynes BF The immune response during acute HIV-1 infection: clues for vaccine development.. Nat Rev Immunol.

[6] Fellay J, Shianna KV, Ge D, Colombo S, Ledergerber B (2007). A Whole-Genome Association Study of Major Determinants for Host Control of HIV-1.. Science (Washington, DC, United States).

[7] Jennes W, Verheyden S, Demanet C, Adje-Toure CA, Vuylsteke B (2006). Cutting Edge: Resistance to HIV-1 Infection among African Female Sex Workers Is Associated with Inhibitory KIR in the Absence of Their HLA Ligands.. Journal of Immunology.

[8] Martin MP, Gao X, Lee JH, Nelson GW, Detels R (2002). Epistatic interaction between *KIR3DS1* and *HLA-B* delays the progression to AIDS.. Nat Genet.

[9] Feng Z, Dubyak GR, Lederman MM, Weinberg A (2006). Cutting edge: human beta defensin 3–a novel antagonist of the HIV-1 coreceptor CXCR4.. J Immunol.

[10] Muench J, Staendker L, Adermann K, Schulz A, Schindler M (2007). Discovery and optimization of a natural HIV-1 entry inhibitor targeting the gp41 fusion peptide.. Cell (Cambridge, MA, United States).

[11] Muench J, Ruecker E, Staendker L, Adermann K, Goffinet C (2007). Semen-Derived Amyloid Fibrils Drastically Enhance HIV Infection.. Cell.

[12] Gasper-Smith N, Crossman DM, Whitesides JF, Mensali N, Ottinger JS (2008). Induction of plasma (TRAIL), TNFR-2, Fas ligand, and plasma microparticles after human immunodeficiency virus type 1 (HIV-1) transmission: implications for HIV-1 vaccine design.. J Virol.

[13] Norris PJ, Pappalardo BL, Custer B, Spotts G, Hecht FM (2006). Elevations in IL-10, TNF-alpha, and IFN-gamma from the earliest point of HIV Type 1 infection.. AIDS Res Hum Retroviruses.

[14] Stacey AR, Norris PJ, Qin L, Haygreen EA, Taylor E (2009). Induction of a striking systemic cytokine cascade prior to peak viremia in acute human immunodeficiency virus type 1 infection, in contrast to more modest and delayed responses in acute hepatitis B and C virus infections.. Journal of Virology.

[15] Fiebig EW, Wright DJ, Rawal BD, Garrett PE, Schumacher RT (2003). Dynamics of HIV viremia and antibody seroconversion in plasma donors: implications for diagnosis and staging of primary HIV infection.. AIDS.

[16] Husebekk A, Permin H, Husby G (1986). Serum amyloid protein A (SAA): An indicator of inflammation in AIDS and AIDS-related complex (ARC).. Scandinavian Journal of Infectious Diseases.

[17] Uhlar CM, Whitehead AS (1999). Serum amyloid A, the major vertebrate acute-phase reactant.. European Journal of Biochemistry.

[18] Vlasova MA, Moshkovskii SA (2006). Molecular interactions of acute phase serum amyloid A: Possible involvement in carcinogenesis.. Biochemistry (Moscow).

[19] Misse D, Yssel H, Trabattoni D, Oblet C, Lo Caputo S (2007). IL-22 participates in an innate anti-HIV-1 host-resistance network through acute-phase protein induction.. J Immunol.

[20] Li W, Savinov AY, Rozanov DV, Golubkov VS, Hedayat H (2004). Matrix metalloproteinase-26 is associated with estrogen-dependent malignancies and targets alpha–1-antitrypsin serpin.. Cancer Research.

[21] Jensen LE, Whitehead AS (1998). Regulation of serum amyloid A protein expression during the acute-phase response.. Biochem J.

[22] Demberg T, Robert-Guroff M (2009). Mucosal immunity and protection against HIV/SIV infection: Strategies and challenges for vaccine design.. International Reviews of Immunology.

[23] He R, Shepard LW, Chen J, Pan ZK, Ye RD (2006). Serum amyloid A is an endogenous ligand that differentially induces IL-12 and IL-23.. J Immunol.

[24] Song C, Hsu K, Yamen E, Yan W, Fock J (2009). Serum amyloid A induction of cytokines in monocytes/macrophages and lymphocytes.. Atherosclerosis.

[25] Blumenthal R, Dimitrov DS (2007). Targeting the sticky fingers of HIV-1.. Cell (Cambridge, MA, United States).

[26] Shapiro L, Pott GB, Ralston AH (2001). Alpha-1-antitrypsin inhibits human immunodeficiency virus type 1.. FASEB Journal.

[27] Nakayama T, Sonoda S, Urano T, Yamada T, Okada M (1993). Monitoring both serum amyloid protein A and C-reactive protein as inflammatory markers in infectious diseases.. Clinical Chemistry.

[28] Shainkin-Kestenbaum R, Zimlichman S, Winikoff Y (1982). Serum amyloid A (SAA) in viral infection: Rubella, measles and subacute sclerosing panencephalitis (SSPE).. Clinical and Experimental Immunology.

[29] Yip TTC, Chan JWM, Cho WCS, Yip TT, Wang Z (2005). Protein chip array profiling analysis in patients with severe acute respiratory syndrome identified serum amyloid A protein as a biomarker potentially useful in monitoring the extent of pneumonia.. Clinical Chemistry.

[30] Cai Z, Cai L, Jiang J, Chang KS, Van Der Westhuyzen DR (2007). Human serum amyloid A protein inhibits hepatitis C virus entry into cells.. Journal of Virology.

[31] Lavie M, Voisset C, Vu-Dac N, Zurawski V, Duverlie G (2006). Serum amyloid A has antiviral activity against hepatitis C virus by inhibiting virus entry in a cell culture system.. Hepatology.

[32] Bristow CL, Patel H, Arnold RR (2001). Self antigen prognostic for human immunodeficiency virus disease progression.. Clinical and Diagnostic Laboratory Immunology.

[33] Hayes VM, Gardiner-Garden M (2003). Are Polymorphic Markers within the alpha-1-Antitrypsin Gene Associated with Risk of Human Immunodeficiency Virus Disease?. Journal of Infectious Diseases.

[34] Anderson ED, Thomas L, Hayflick JS, Thomas G (1993). Inhibition of HIV-1 gp160-dependent membrane fusion by a furin-directed alpha–1-antitrypsin variant.. Journal of Biological Chemistry.

[35] McNeely TB, Shugars DC, Rosendahl M, Tucker C, Eisenberg SP (1997). Inhibition of human immunodeficiency virus type 1 infectivity by secretory leukocyte protease inhibitor occurs prior to viral reverse transcription.. Blood.

[36] Congote LF (2006). The C-terminal 26-residue peptide of serpin A1 is an inhibitor of HIV-1.. Biochemical and Biophysical Research Communications.

[37] Congote LF (2007). Serpin A1 and CD91 as host instruments against HIV-1 infection: Are extracellular antiviral peptides acting as intracellular messengers?. Virus Research.

[38] Holtz B, Cuniasse P, Boulay A, Kannan R, Mucha A (1999). Role of the S1' subsite glutamine 215 in activity and specificity of stromelysin-3 by site-directed mutagenesis.. Biochemistry.

[39] Liu Z, Zhou X, Shapiro SD, Shipley JM, Twining SS (2000). The serpin alpha-1-proteinase inhibitor is a critical substrate for gelatinase B/MMP-9 in vivo.. Cell.

[40] Mastroianni CM, Liuzzi GM (2007). Matrix metalloproteinase dysregulation in HIV infection: implications for therapeutic strategies.. Trends in Molecular Medicine.

[41] Mellanen L, Lähdevirta J, Tervahartiala T, Meurman JH, Sorsa T (2006). Matrix metalloproteinase-7, -8, -9, -25, and -26 and CD43, -45, and -68 cell-markers in HIV-infected patients' saliva and gingival tissue.. Journal of Oral Pathology and Medicine.

[42] Zhang Z, Winyard PG, Chidwick K, Murphy G, Wardell M (1994). Proteolysis of human native and oxidised alpha–1-proteinase inhibitor by matrilysin and stromelysin.. Biochimica et Biophysica Acta - General Subjects.

[43] Cole AM, Shafer WM (2006). Innate host defense of human vaginal and cervical mucosae.. Current Topics in Microbiology and Immunology.

[44] Cole AM, Cole AL (2008). Antimicrobial polypeptides are key anti-hiv-1 effector molecules of cervicovaginal host defense.. American Journal of Reproductive Immunology.

[45] Burgener A, Boutilier J, Wachihi C, Kimani J, Carpenter M (2008). Identification of differentially expressed proteins in the cervical mucosa of HIV-1-resistant sex workers.. Journal of Proteome Research.

[46] Xu D, Suenaga N, Edelmann MJ, Fridman R, Muschel RJ (2008). Novel MMP-9 substrates in cancer cells revealed by a label-free quantitative proteomics approach.. Molecular and Cellular Proteomics.

[47] Batycka M, Inglis NF, Cook K, Adam A, Fraser-Pitt D (2006). Ultra-fast tandem mass spectrometry scanning combined with monolithic column liquid chromatography increases throughput in proteomic analysis.. Rapid Communications in Mass Spectrometry.

[48] Wessel D, Flugge UI (1984). A method for the quantitative recovery of protein in dilute solution in the presence of detergents and lipids.. Analytical Biochemistry.

