# Supplementary material for: Elevation of Intact and Proteolytic Fragments of Acute Phase Proteins Constitutes the Earliest Systemic Antiviral Response in HIV-1 Infection
Source: PLoS Pathog. 2010 May 6;6(5):e1000893. doi: 10.1371/journal.ppat.1000893 (PMC2865525; doi:10.1371/journal.ppat.1000893)
Supplement: Figure S1 — Identification of A-SAA and complement C3 derived peptides by tandem mass spectrometry. (A). LC-MS/MS analysis of plasma donor sample 64012 time point T0+4 identified precursor ion 1108.6 Da [M+2H]2+ that corresponded to peptide 960–979 derived from complement C3 (Swissprot accession nr. P01024). Identified b- and y- fragment ions are indicated. (B). LC-MS/MS analysis of plasma donor sample 64012 time point T0+13 revealed precursor ion mass of 1090.4 Da [M+2H]2+ that corresponded to peptide 86–105 derived from A-SAA (Swissprot accession nr. P02735). (0.21 MB PDF) [file ppat.1000893.s001.pdf]

A

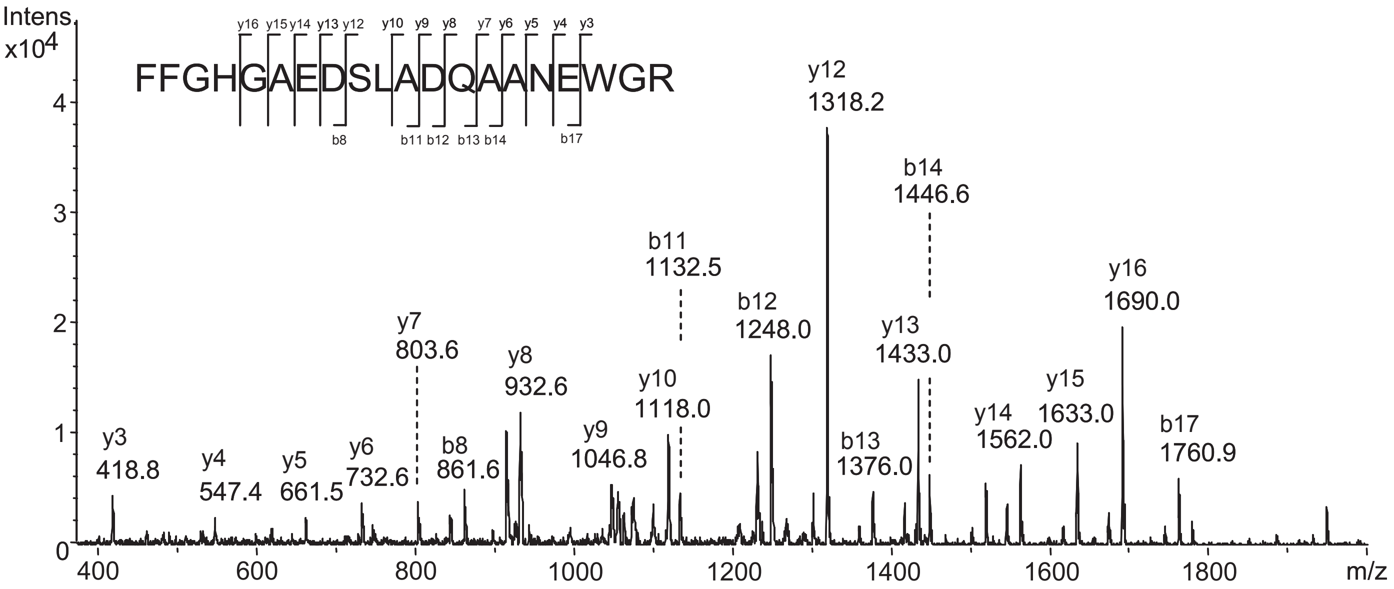

A-SAA peptide 86-105 (P02735)
Precursor ion mass observed 1089.8 Da $[M+2H]^{2+}$, M calculated: 2178.8 Da

B

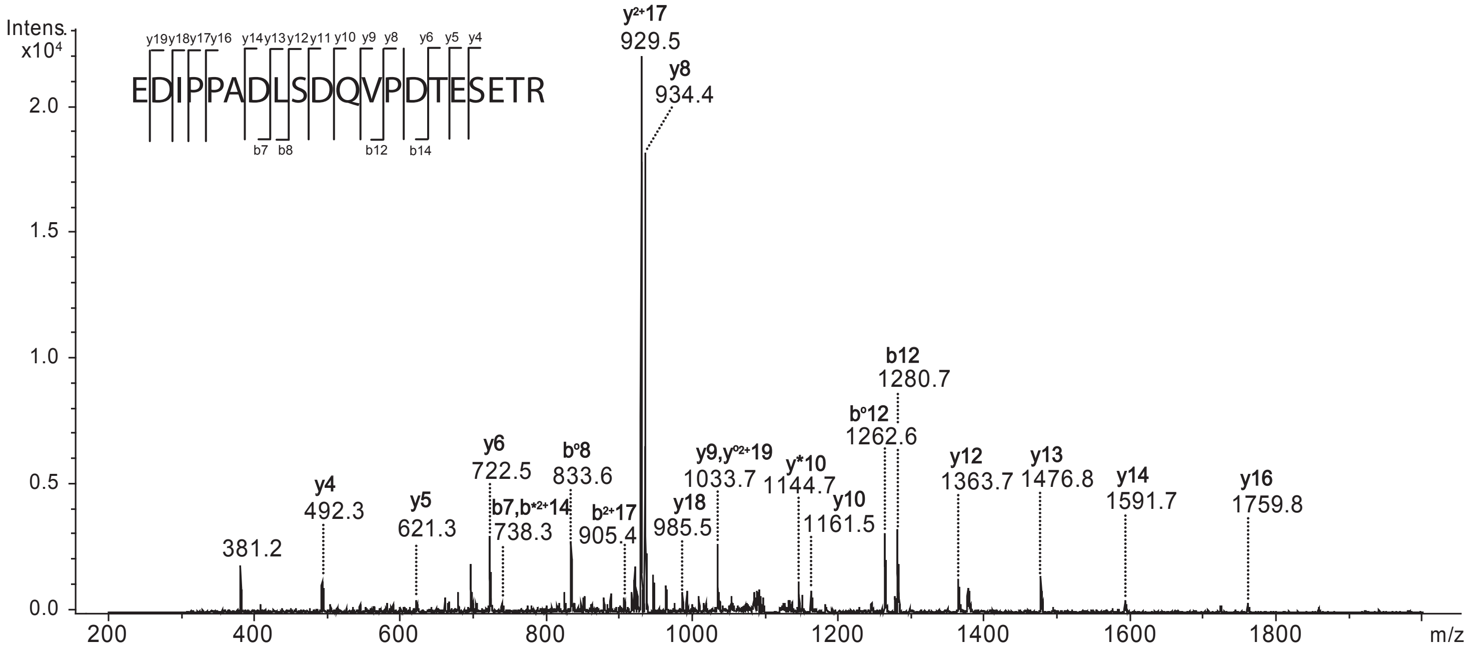

Complement C3 peptide 960-979 (P01024)
Precursor ion mass observed 1108.6 Da $[M+2H]^{2+}$, M calculated: 2213.0 Da
