# Supplementary figures and images for: Elevation of Intact and Proteolytic Fragments of Acute Phase Proteins Constitutes the Earliest Systemic Antiviral Response in HIV-1 Infection
Source: PLoS Pathog. 2010 May 6;6(5):e1000893. doi: 10.1371/journal.ppat.1000893 (PMC2865525; doi:10.1371/journal.ppat.1000893)

**A**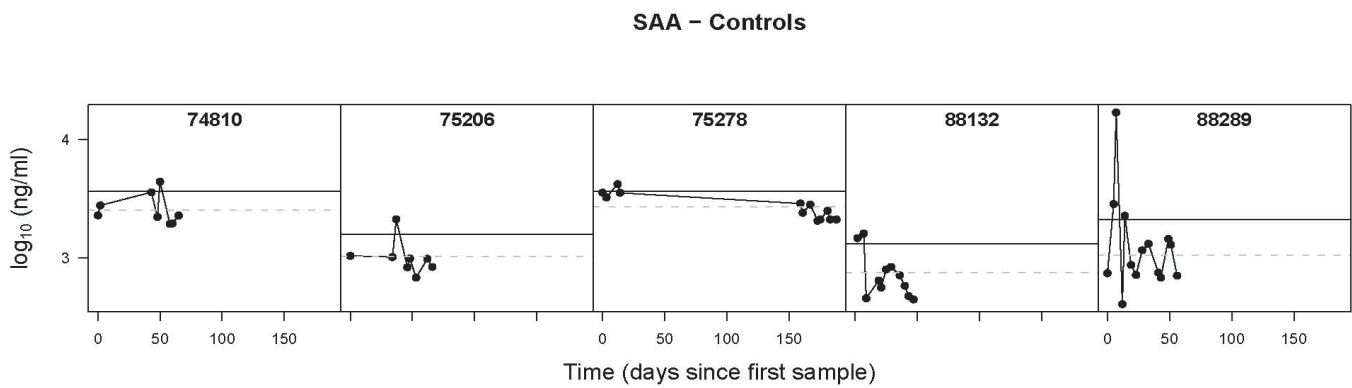**B**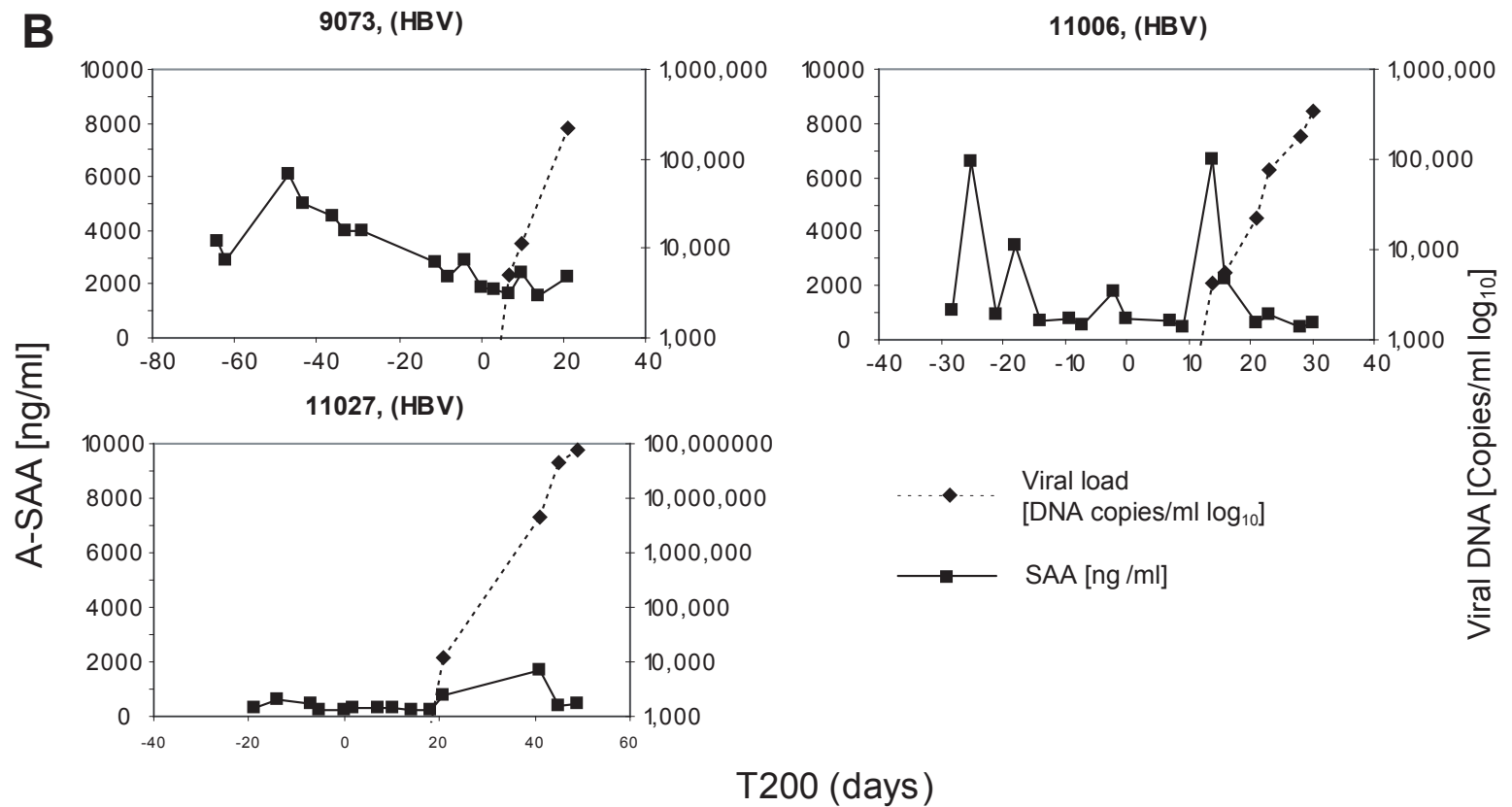**C**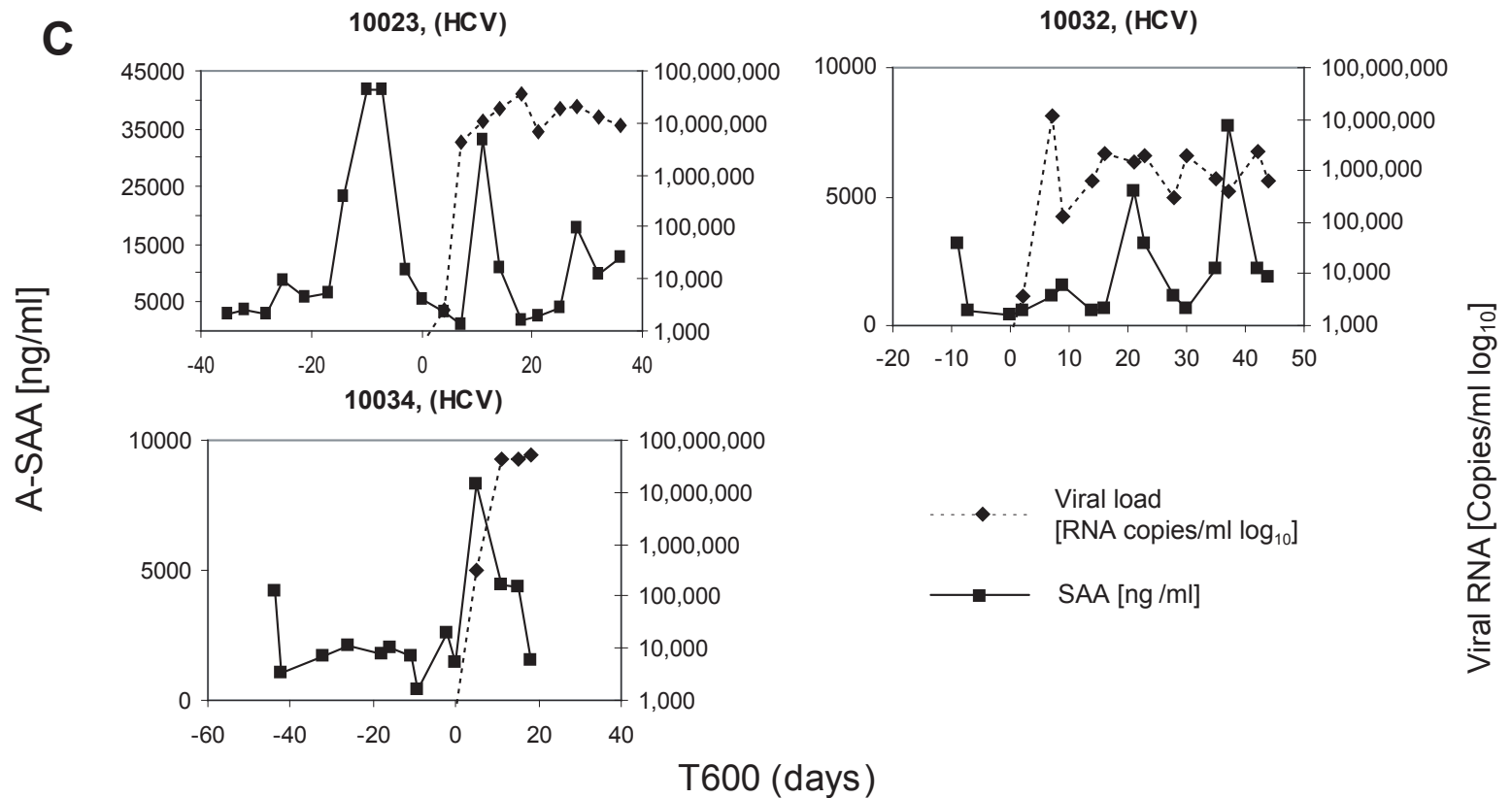

Supplement: Figure S2 — A-SAA levels as measured by ELISA in plasma panels from 5 control plasma donors, 3 plasma donors acquiring HBV infection and 3 donors acquiring HCV infection. (A). A-SAA levels in plasma panels from five control non-HIV-infected plasma donors. The solid circles show A-SAA protein levels. Subject-specific background levels of A-SAA (dotted grey lines) and the 90% prediction interval (threshold for defining a significant elevation, black lines) were calculated as described in the methods section. (B). A-SAA levels in plasma panels from three HBV-infected donors. In each graph, time is plotted relative to T200 (first time point where DNA levels are above 200 copies ml−1). A-SAA protein levels are indicated by black squares and solid black lines and HBV DNA levels by black triangles and dotted black lines. (C). A-SAA levels in plasma panels from three HCV-infected donors. In each graph, time is plotted relative to T600 (first time point where RNA levels are above 600 copies ml−1). A-SAA protein levels are indicated by black squares and solid black lines and HCV RNA levels by black triangles and dotted black lines. (0.33 MB PDF) [file ppat.1000893.s002.pdf]

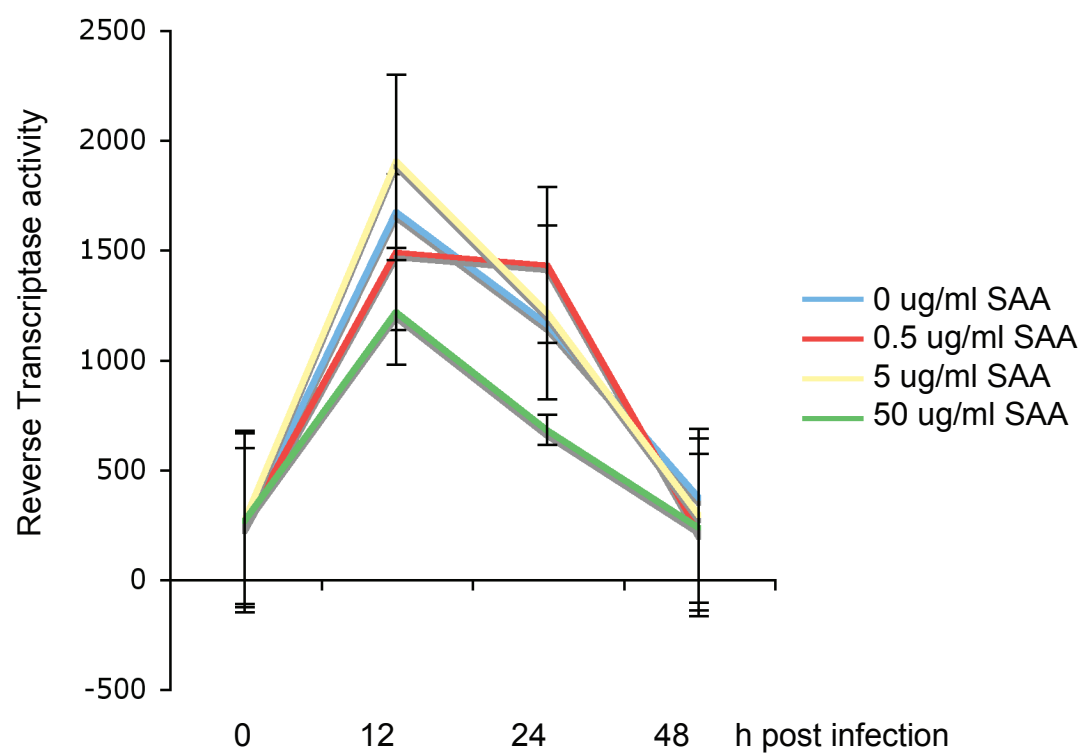

Supplement: Figure S3 — Effect of A-SAA on HIV infection of MDDCs. MDDCs were incubated with the indicated concentrations of A-SAA and infected with R5 virus produced from the infectious molecular HIV clone pNL4.3-Bal.ecto. Reverse transcriptase levels were determined at 12, 24 and 48 h. Results are the average of values from four sets of MDDCs generated from four separate buffy coats. The error bars indicate 1 standard error above and below the mean. (0.13 MB PDF) [file ppat.1000893.s003.pdf]
