# Supplementary material for: Elevation of Intact and Proteolytic Fragments of Acute Phase Proteins Constitutes the Earliest Systemic Antiviral Response in HIV-1 Infection
Source: PLoS Pathog. 2010 May 6;6(5):e1000893. doi: 10.1371/journal.ppat.1000893 (PMC2865525; doi:10.1371/journal.ppat.1000893)
Supplement: Text S1 — Supporting Information (0.03 MB DOC) [file ppat.1000893.s005.doc]

**Supporting Information**

**Reagents, antibodies and chemicals**

The following materials and reagents were used in this study: NuPAGE 4-12% Bis-Tris Gels (Invitrogen, #NP0321), Novex 16% Tricine Gels (Invitrogen, #EC66952), Immobilon P membrane (Millipore, #IPVH0010), ECL Plus Western Blotting detection system (GE Healthcare, #RPN2132), Kodak Biomax XAR film (Sigma-Aldrich, # F5513), MB-WAX Profiling kit (Bruker daltonics, #223988), Isolute C18 cartridges (Biotage, #220-0020-A), Human SAA ELISA kit (Abazyme, #EL10015), A-SAA mouse mAb (Abcam, #ab687), SAA4 (C-SAA) mouse pAb (Abnova, #6291), α1-antitrypsin rabbit pAb (Aviva, #AVARP00015), ceruloplasmin goat pAb (Bethyl, #A80-124A), antithrombin III sheep pAb (Biodesign, #K950085), Goat anti-mouse IgG-HRP (Dako, #P0447), Goat anti-rabbit IgG-HRP (Dako, #P0448), Rabbit anti-sheep IgG-HRP (Dako, #P0163), Rabbit anti-goat IgG-HRP (Dako, #P0160), recombinant MMP-7 (Calbiochem, #444270), α1-antitrypsin (Sigma, #A6150), A-SAA (MBL International, #JM-4324), SAA4 protein (Abcam, #ab40603), Ceruloplasmin (Calbiochem, #239799). Synthetic VIRIP LEAIPMSIPPEVKFNKPFVF was synthesized by Fmoc-based solid phase peptide synthesis on an automated synthesizer (Advanced ChemTech). Rabbit polyclonal anti-VIRIP antisera were generated using a synthetic peptide. A C-terminal cysteine was added for the coupling to KLH carrier protein (Pierce, IL, USA) according to the manufacturer’s instructions. Anti-VIRIP polyclonal antiserum was raised in rabbits by CoVal Ab (Cambridge, UK).

**Western blot analysis**

Freshly thawed plasma samples were aliquoted (2 μl each), diluted with reducing SDS-PAGE sample buffer (18 μl) and heated at 95°C for 10 min. Samples (2 μl, corresponding to 0.2 μl plasma per well) were separated by 15% SDS-PAGE followed by overnight transfer onto PVDF membrane and immunoblotting using specific antibodies (anti-A-SAA primary mAb 1:4’000, secondary Ab 1:10’000; anti-C-SAA primary pAb 1:2’000, secondary Ab 1:10’000). Detection was carried out using enhanced chemo luminescence detection (GE Healthcare, Little Chalfont, United Kingdom) and recorded on photographic film (Kodak, Rochester, NY).

**Sample preparation for analysis by mass spectrometry**

Plasma donor samples were fractionated using weak anion exchange (WAX) magnetic beads (Bruker Daltonics, Bremen, Germany) according to the manufacturer’s recommendation. In brief, 10 μl of bead suspension was used per sample. Supernatants were removed by retaining the beads with a magnet, followed by addition of activation solution (10 μl each) and incubation at room temperature for 1 min. After removal of supernatant, activated beads were mixed with 10 μl plasma which had been previously aliquoted and digested with sequencing grade trypsin (Promega, Madison, WI) for 16 h at 37°C at a final concentration of 20 ng μl^-1^. After incubation for 5 min, beads were washed three times with 100 μl binding buffer, pH 9.0 and finally eluted with 10 μl elution buffer. Samples were kept at -20^o^C prior to analysis by MALDI-TOF or LC-MS/MS.

As an alternative fractionation method, plasma samples were thawed at room temperature and aliquoted (10 μl each). A fresh solution of trypsin in 100 mM ammonium bicarbonate was prepared and added to plasma at a final concentration of 20 ng μl^-1^ followed by incubation at 37°C for 16 h. Aliquots were diluted with 2% acetonitrile, 0.1% formic acid (500 μl each) and loaded onto a freshly equilibrated C18 solid phase extraction (SPE) column (SepPack, Waters, Milford, MA, USA) using a vacuum manifold (Kinesis, UK). Unbound material was washed away with buffer containing 2% acetonitrile, 0.1% formic acid in H_2_O (5 ml each) and bound peptides were eluted with 80% acetonitrile, 0.1% formic acid in H_2_O. Eluates were concentrated overnight (16 h) under reduced pressure and lyophilized material was resuspended by sonication for 5 min in 10 μl of 2% acetonitrile, 0.1% formic acid in water. Samples were stored at -20^o^C until analysis.

**Alternative HIV-1 infection assay (Fig. S3)**

Viral stocks were generated by transfection of 293T cells with calcium-phosphate co-precipitated proviral plasmid encoding for pNL4.3-Bal.ecto (R5). MDDCs were generated from buffy coats (National Blood Centre (UK)) by adherence and culture in IL-4 and GM-CSF. MDDC preparations analyzed were more than 98% pure. Inhibitors were preincubated with cells for 2 h and infection (MOI 0.01) was carried out for 12, 24 and 48 h at 37°C in the presence of inhibitor. After this time period supernatants were harvested and an HIV-1 reverse transcriptase assay (GE Healthcare, Amersham, UK) was used for quantitation.
